# Supplementary figures and images for: CCNE1 Is a Putative Therapeutic Target for ARID1A-Mutated Ovarian Clear Cell Carcinoma
Source: Int J Mol Sci. 2021 May 30;22(11):5869. doi: 10.3390/ijms22115869 (PMC8198755; doi:10.3390/ijms22115869)

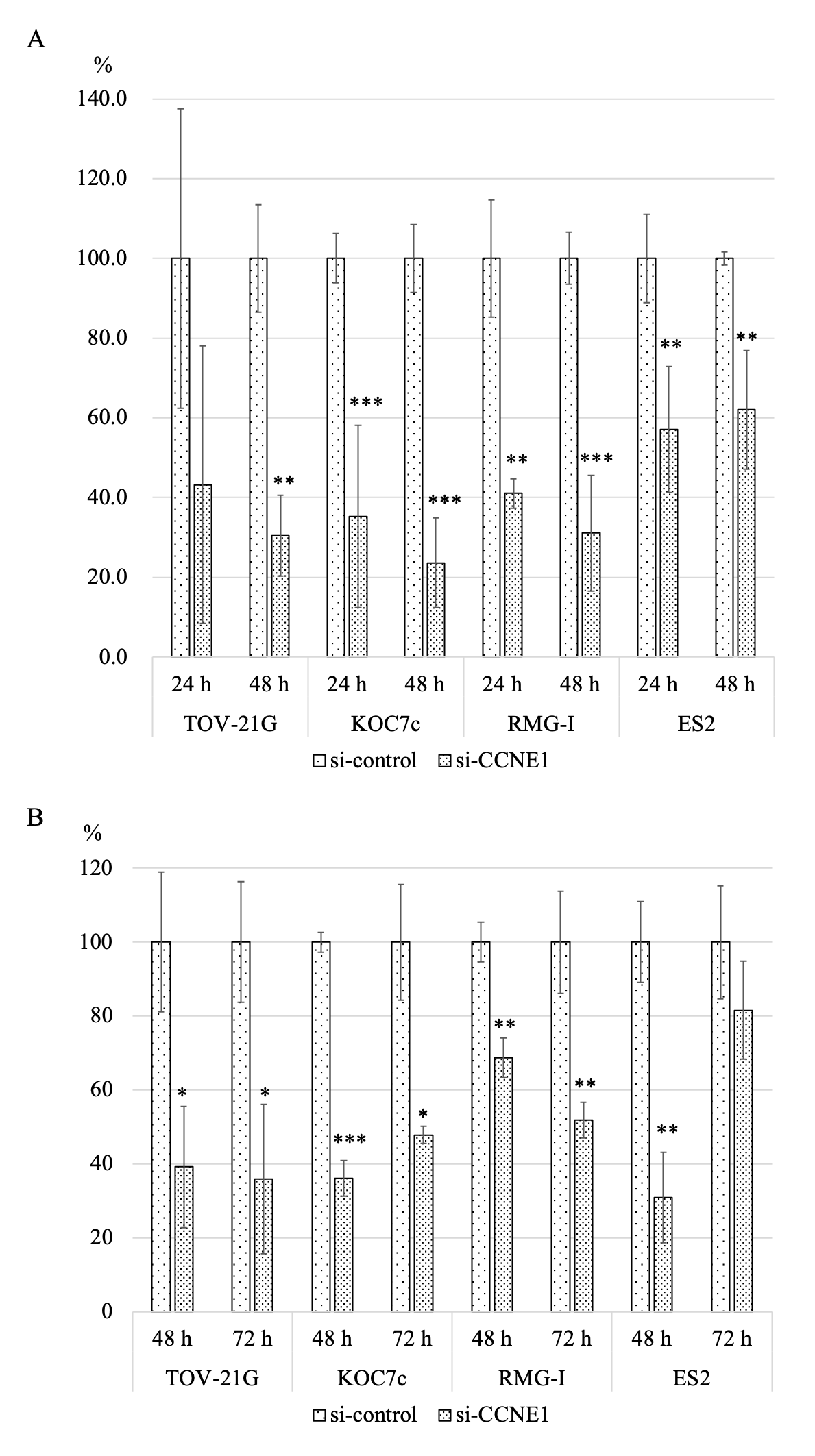

Supplement: Supplementary file 1 [file ijms-22-05869-s001.zip › Supplementary figures/Figure S1.tif]

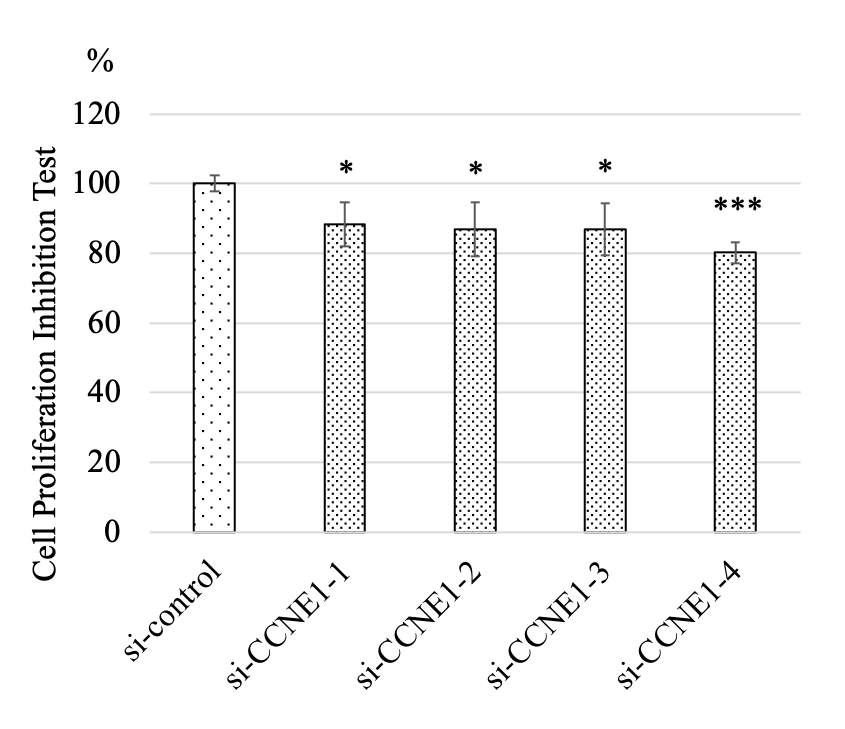

Supplement: Supplementary file 1 [file ijms-22-05869-s001.zip › Supplementary figures/Figure S2.tif]

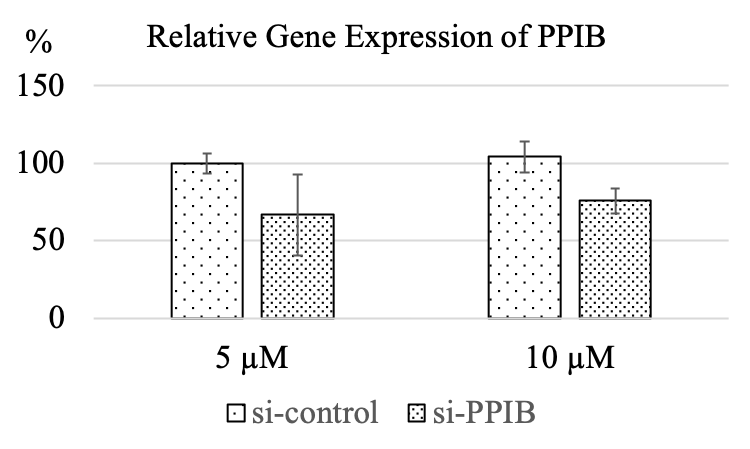

Supplement: Supplementary file 1 [file ijms-22-05869-s001.zip › Supplementary figures/Figure S3.tif]
